# Myeloid-Derived Suppressor Cells: A Multifaceted Accomplice in Tumor Progression

**DOI:** 10.3389/fcell.2021.740827

**Published:** 2021-12-23

**Authors:** Jia-Nan Cheng, Yi-Xiao Yuan, Bo Zhu, Qingzhu Jia

**Affiliations:** ^1^ Department of Oncology, Xinqiao Hospital, Third Military Medical University, Chongqing, China; ^2^ Chongqing Key Laboratory of Immunotherapy, Chongqing, China; ^3^ Department of Thoracic Surgery, The Third Affiliated Hospital of Kunming Medical University, Kunming, China

**Keywords:** MDSC, Treg, EMT, angiogenesis, immunotherapy

## Abstract

Myeloid-derived suppressor cell (MDSC) is a heterogeneous population of immature myeloid cells, has a pivotal role in negatively regulating immune response, promoting tumor progression, creating pre-metastases niche, and weakening immunotherapy efficacy. The underlying mechanisms are complex and diverse, including immunosuppressive functions (such as inhibition of cytotoxic T cells and recruitment of regulatory T cells) and non-immunological functions (mediating stemness and promoting angiogenesis). Moreover, MDSC may predict therapeutic response as a poor prognosis biomarker among multiple tumors. Accumulating evidence indicates targeting MDSC can reverse immunosuppressive tumor microenvironment, and improve therapeutic response either single or combination with immunotherapy. This review summarizes the phenotype and definite mechanisms of MDSCs in tumor progression, and provide new insights of targeting strategies regarding to their clinical applications.

## 1 Introduction

Myeloid cells, which are derived from hematopoietic stem cells, have diverse functions such as protect human body against infection and help tissue repair. Mature myeloid cells, including macrophages, neutrophils, eosinophils, and basophils, are a key component of the innate immune system. However, the majority of these pro-inflammatory cells can be re-educated to become pro-tumor cells when tumorigenesis occurs. Significantly increased immature myeloid cells have been observed in the bone marrow and peripheral blood of patients with cancer (Yang et al., 2004). These immature myeloid cells are called myeloid-derived suppressor cells (MDSCs) because of their capacity to suppress the anti-tumor immune response (Talmadge and Gabrilovich, 2013).

The existence of enriched MDSCs have been shown to be related with poor prognosis for multiple types of cancer (Jiang et al., 2015; Tian et al., 2015). Various pro-tumor mechanisms have been reported: MDSCs suppress the proliferation and cytolysis of T cells (Chen et al., 2017) and promote the recruitment and expansion of Treg cells (Serafini et al., 2008). MDSCs are also involved in angiogenesis (Lee et al., 2005) and the maintenance of stemness (Wang et al., 2019a). However, in most studies the role of MDSCs have been studied independently and generate fragmented information. The limitations hinder our understanding of MDSCs in tumor microenvironment and optimize anti-tumor treatment.

In this review, we systematically summarize the characteristics and definite mechanisms of MDSCs in tumor progression and metastasis, highlight the role of MDSCs in clinical treatment, and further discuss the potential strategies to provide new insights regarding their clinical applications.

## 2 Definition of MDSCs

**FIGURE 1:**
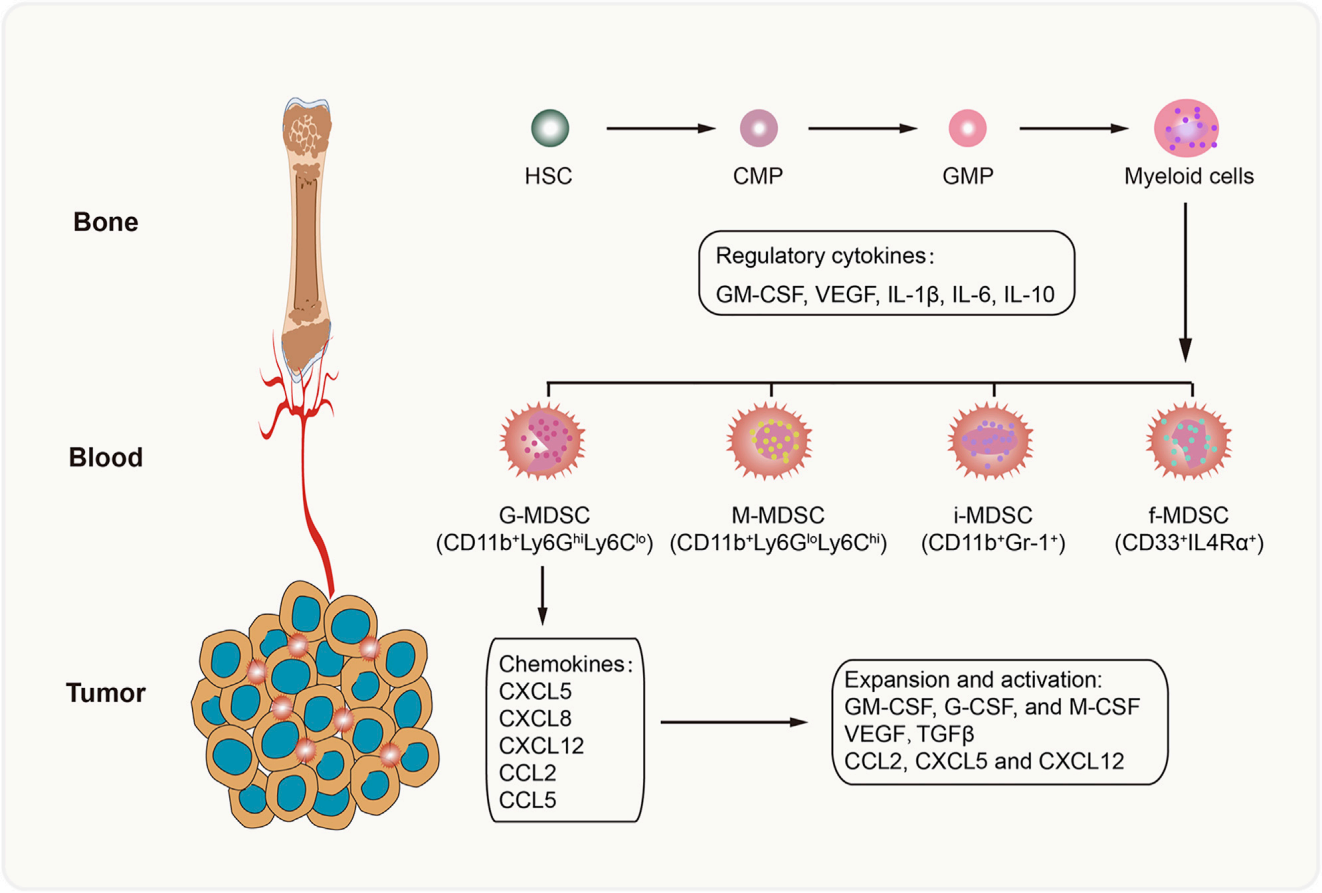
Development of MDSCs. MDSCs are derived from hematopoietic stem cells (HSC) in bone marrow, and differentiate into myeloid cells step by step. In the pathological conditions, a series of cytokines (GM-CSF, VEGF, IL-1β, IL-6, and IL-10) promote MDSC generation. Then suppressive MDSCs are recruited into TME by tumor-derived chemokines, such as CXCL5, CXCL8, CXCL12, CCL2, and CCL5. Further, the favorable TME accelerates the activation, survival and expansion of MDSCs. CMP, common myeloid progenitor; GMP, granulocyte and macrophage progenitor.

MDSCs comprise a heterogeneous population of immature myeloid cells that are divided into different subsets based on morphology and surface markers. In mice, MDSCs are mainly categorized into two groups: granulocytic MDSCs (G-MDSCs; or polymorphonuclear MDSCs [PMN-MDSCs]) and monocytic (M) MDSCs (M-MDSCs) (Zhao et al., 2016). Other subpopulations found in humans are immature MDSCs (i-MDSCs) (Mandruzzato et al., 2016) and fibrocytic MDSCs (f-MDSCs) (Zoso et al., 2014). G-MDSCs, which are characterized as CD11b^+^Ly6G^hi^Ly6C^lo^ cells, mainly produce reactive oxygen species (ROS) to suppress T-cell function (Tacke et al., 2012). The M-MDSCs referred to as CD11b^+^Ly6G^lo^Ly6C^hi^ cells can inhibit the immune response by producing nitric oxide (NO), arginase 1 (Arg-1), and IL-10 (Maenhout et al., 2014). The f-MDSC subset, which is characterized by the expression of CD33^+^IL4Rα^+^, attenuates T-cell proliferation via indoleamine 2-3 dioxygenase (IDO) secretion and promotes regulatory T (Treg) cell expansion (Zoso et al., 2014). Nevertheless, the phenotype and function of i-MDSCs have not been determined (Figure 1).

Although the generation of MDSCs might be explained by the interrupted development of normal myelopoiesis and abnormal expansion of immature myeloid cells (Netherby and Abrams, 2017), the underlying mechanisms are unclear. Several cytokines and growth factors secreted by tumor cells and immune cells have been shown to promote the accumulation and expansion of MDSCs by activating the JAK/STAT1 and JAK/STAT3 signaling pathways (Fleming et al., 2018), including granulocyte-macrophage colony-stimulating factor (GM-CSF), vascular endothelial growth factor (VEGF), transforming growth factor-β (TGF-β), interleukin-6 (IL-6), and interleukin-10 (IL-10). Moreover, these cells can recruit MDSCs into the tumor microenvironment (TME) by releasing certain chemokines, such as CXCL5, CXCL8, CXCL12, CCL2, and CCL5 (Ozga et al., 2021).

## 3 Contribution of MDSCs to Tumor Progression

### 3.1 Immunosuppressive Functions of MDSCs on Tumor Progression

**FIGURE 2:**
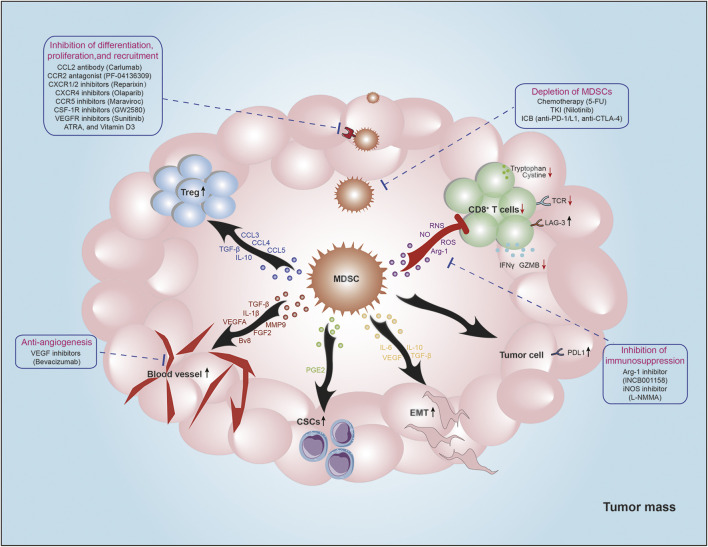
The roles of MDSCs in TME. MDSCs are potent suppressors of antitumor immunity. MDSCs decrease TCR expression and impair cytotoxic T cell function via producing Arg-1, NO, ROS, IL-10, and TGF-β. Moreover, MDSCs promote Tregs recruitment by secreting certain chemokines (CCL3, CCL4, and CCL5), and increase Tregs function via producing IL-10 and TGF-β. Additionally, MDSCs increase PD-L1 expression of tumor cells to mediate immune evasion. MDSCs also play non-immunological functions. MDSCs mediate EMT and stemness of tumor cells by producing VEGF, PEG2, IL-6, IL-10, and TGF-β. And MDSCs could induce angiogenesis via secreting VEGFA, FGF2, Bv8, TGF-β, IL-1β, and MMP9 to promote tumor metastasis. Strategies targeting MDSCs for cancer treatment are summarized around the main figure.

MDSCs are potent suppressors of T-cell activation and function through various mechanisms, including the expression of ligands of negative immune checkpoint regulators, secretion of immunosuppressive cytokines, disruption of amino acid metabolism. Moreover, MDSCs also play a critical role in establishment and maintenance of immunosuppressive microenvironment by interacting with other immune cells, including nature killer cells (NK cells), dendritic cells (DCs), macrophages, and Treg cells (Figure 2).

#### 3.1.1 Suppression of T Cells by Expression of Ligands of Negative Immune Checkpoint Regulators

MDSCs are the main suppressive cells in the anti-tumor immune response; their main role is regulating the quantity and functions of T cells. Tumor-infiltrating MDSCs express high levels of Fas ligand (Fas-L) to induce apoptosis of CD8^+^ T cells by binding to the Fas receptor (Rashid et al., 2021). A previous study found that PMN-MDSCs rather than M-MDSCs could specially induce CD8^+^ T cells apoptosis through Fas-FasL axis and result in local immune suppression (Zhu J et al., 2017). And activated CD8^+^ T cells might also lead to MDSC apoptosis via the Fas-FasL axis (Sinha et al., 2011). Therefore, inducing apoptosis between MDSCs and T cells might result in reciprocal actions. Additionally, MDSCs in TME highly express several ligands of negative immune checkpoint regulators, programmed cell death protein ligand 1 (PD-L1) (Lu et al., 2016) and Galectin-9 (Dardalhon et al., 2010), which bind to programmed cell death protein 1 (PD-1) or T-cell immunoglobulin and mucin domain-containing protein 3 (TIM3) to induce effector T-cell anergy.

#### 3.1.2 Suppression of T Cells by Secretion of Immunosuppressive Cytokines

In response to complex tumor microenvironment, MDSCs develop formidable plasticity, including cytokines, chemokines, and growth factors which are responsible for the suppressive capacity (Lechner et al., 2010). These products are potential therapeutic targets for inhibition of MDSC.

##### IL-10

Increased level of IL-10 has been reported in many cancers. In 2011, Hart *et al* have identified MDSCs as the predominant producers of IL-10 in a mouse model of ovarian cancer (Hart et al., 2011). They also found that IL-10 produced by MDSCs increased lymphocyte activation gene 3 (LAG-3) expression and decreased interferon (IFN)-γ secretion by T cells (Hart et al., 2011). MDSCs can activate the immunosuppressive capacity through IL-10 in pathological conditions and contribute to disease progression (Yaseen et al., 2020). The conclusion was further supported by a previous finding that IL-10 level was significantly reduced by MDSCs depletion in ovarian cancer-bearing mice (Hart et al., 2009). It has been revealed that IL-10 level was positively correlated with MDSCs expansion and tumor progression in patients with anaplastic thyroid cancer, ovarian cancer, gastric cancer and no-small cell lung cancer (Suzuki et al., 2013; Li et al., 2015; Pogoda et al., 2016; Wu et al., 2017). Further studies reported that increased production of IL-10 by MDSCs could inhibit the production of IL-2, IL-12 and IFN-γ by CD4^+^ or CD8^+^ T cells, which lead to their impaired proliferation and anti-tumor immunity (Vuk-Pavlovic et al., 2010; Li et al., 2015). Interestingly, IL-10/IL-10 receptor signaling is also critical for phenotypic and functional maintenance of MDSCs (Bah et al., 2018). It has been reported that blocking IL-10/IL-10R could decrease the infiltration of MDSCs in ascites of ovarian cancer-bearing mice (Lamichhane et al., 2017). Moreover, IL-10 has been shown to strength the immunosuppressive functions of MDSCs by upregulating the expression of ARG-1 and programmed cell death protein 1 (PD-1) (Xiu et al., 2015; Lamichhane et al., 2017). These results indicated that there was positive feedback between MDSCs and IL-10. In addition, IL-10 is also involved in the differentiation and expansion of Treg cells (Heo et al., 2010). Researchers found that IL-10 treatment could induce Foxp3 expression of CD4^+^ T cell population and increase the number of Treg cells. Further studies found that histone deacetylase 11 (HDAC11) and alarmin high mobility group box 1 (HMGB1) are key negative regulators of IL-10 transcription in MDSCs (Villagra et al., 2009; Parker et al., 2014), indicating a promising therapeutic strategy for enhancing anti-tumor immunity.

##### TGF-β

Increased TGF-β production in MDSCs has been identified in various tumor types. Chikamatsu *et al* reported that CD14^+^ HLA-DR^−^ MDSCs produced high level of TGF-β to inhibit T cell proliferation and IFN-γ production in squamous cell carcinoma of the head and neck. And blocking TGF-β with antibody could partially the immunosuppressive functions of MDSCs (Chikamatsu et al., 2012; Novitskiy et al., 2012). Moreover, in a mouse model with specific deletion of Tgfbr2 in myeloid cells, the suppressive function of CD11b^+^Gr1^+^ MDSCs was found to decreased significantly (Novitskiy et al., 2012). The TGF-β family of proteins is a potent immune regulator that can inhibit proliferation, differentiation, activation, and cytotoxicity of effector T cells. It has been reported that TGF-β blocked naïve T cells differentiated into Th1 cells, which is the most important subset to mediate anti-tumor response (Sad and Mosmann 1994). Further studies revealed that the impaired differentiation might due to the silence of the expression of two Th1 master transcription factors, TBET and STAT4 (Gorelik et al., 2002; Lin et al., 2005). Chen and colleagues found that TGF-β inhibited T cell activation by dampening the initial Ca^2+^ influx triggered T Cell Receptor (TCR) stimulation (Chen et al., 2003). In addition to inhibiting differentiation and activation, TGF-β blocks T cell proliferation and effector functions. TGF-β could decrease IL-2 expression of T cells to inhibit proliferation by Smad3 signaling (McKarns et al., 2004). Thomas and Massague revealed that the TGF-β/Smad pathway could directly bind to their promoter regions to downregulate the expression of granzyme B and IFN-γ (Thomas and Massague, 2005), which are responsible for cytotoxic T-lymphocyte-mediated tumor cytotoxicity. Moreover, depletion of Tgfbr2 promoted the expression of receptor KLRG1 and production of granzyme-B and IFN-γ (Zhang and Bevan, 2012). Furthermore, TGF-β has a significant role in the generation and expansion of Treg cells with powerful immunosuppressive potential (Wan and Flavell 2008). Further evidence demonstrated that TGF-β could induce CD25 and Foxp3 expression to convert native CD4^+^ T cells to Treg cells (Fu et al., 2004).

##### Chemokines

In addition to releasing immunosuppressive cytokines, MDSCs can produce high levels of CCL3, CCL4, and CCL5, which attract Treg cells into the TME via preferential expression of CCR5 on the Treg cell surface (Schlecker et al., 2012).

#### 3.1.3 Suppression of T Cells by Disruption of Amino Acid Metabolism

As previously mentioned, MDSCs produce Arg-1, inducible nitric oxide synthase (iNOS), and ROS to impair T-cell-mediated immune responses. The underlying mechanisms have been determined by the exhaustion of certain amino acids and the generation of free radicals.

##### Arg1

Arg-1 hyperproduction of MDSCs in human cancer was first reported by Zea et al., in 2005 (Zea et al., 2005); they found that increased Arg activity was limited to a specific subset of CD11b^+^CD14^−^CD15^+^ cells in the peripheral blood of patients with metastatic renal cell carcinoma instead of macrophages or dendritic cells described in mouse models. Arg-1 utilizes L-arginine to produce urea, causing L-arginine deficiency in the TME. L-arginine deficiency leads to cell cycle arrest during the G0-G1 phase of T cells by upregulating cyclin D3 and cyclin-dependent kinase 4 (cdk4), resulting in decreased phosphorylation of Rb protein and low expression and binding of E2F1 (Rodriguez et al., 2007; Rodriguez et al., 2009). The absence of L-arginine also appears to downregulate T-cell receptor (TCR) expression by decreasing CD3ζ chain biosynthesis to induce T-cell dysfunction (Rodriguez et al., 2002).

##### iNOS and ROS

Similarly, L-arginine is also a substrate for iNOS to produce NO (Lee et al., 2003). In addition to depleting L-arginine, NO can suppress T cells by inhibiting JAK3/STAT5 activation (Bingisser et al., 1998), decreasing MHC class II molecule expression (Harari and Liao 2004), and inducing T-cell apoptosis (Vig et al., 2004). Additionally, NO can cooperate with ROS to form the reactive nitrogen species (RNS) peroxynitrite (Gabrilovich et al., 2012), which nitrates tyrosine residue in proteins involved in T-cell function. Lu et al. reported that lymphocyte-specific protein tyrosine kinase (LCK) was nitrated at Tyr394 by MDSCs as an initiating tyrosine kinase in the TCR signaling cascade (Feng et al., 2018). LCK nitration reduced IL-2 production to inhibit the proliferation and activation of T cells. Moreover, RNS nitrates the tyrosine of TCR to modify its conformational flexibility and affect its interaction with MHC class I molecules, thus causing the decreased response of CD8^+^ T cells to antigen-specific stimulation (Nagaraj et al., 2007). Additionally, RNS induces the nitration of CCL2 chemokines to inhibit antigen-specific cytotoxic T-cell trafficking into the tumor (Molon et al., 2011). Upregulated ROS have been identified in the MDSCs of many tumors (Ohl and Tenbrock 2018). Other studies have found that MDSCs with decreased ROS production failed to inhibit IFN-γ secretion and proliferation of antigen-specific CD8^+^ T cells (Corzo et al., 2009). In addition to direct toxicity, ROS might be involved in TCR CD3ζ expression (Otsuji et al., 1996), which limits the activation and expression of IFN-γ in T cells.

##### IDO1

The abnormal metabolisms of tryptophan and cystine induced by MDSCs also mediate T-cell suppression. MDSCs highly express indoleamine 2,3-dioxygenase 1 (IDO1), which is an enzyme that hydrolyzes tryptophan along the kynurenine pathway (Yu et al., 2013). A shortage of tryptophan causes T-cell proliferation arrest during the G1 phase of the cell cycle by stimulating general control nonderepressible 2 (GCN2) activation (Munn et al., 2005). Furthermore, GCN2 activation could reduce the expression of the TCR CD3ζ chain to inhibit antigen presentation (Fallarino et al., 2006). Metz et al. reported that the IDO-mediated catabolism of tryptophan also inhibited the mammalian target of rapamycin (mTOR) and protein kinase C (PKC), along with autophagy of effector T cells (Metz et al., 2012). Furthermore, IDO1 promotes the expansion of Treg cells and enhances their immunosuppressive functions to mediate immune tolerance (Curti et al., 2007). A recent study found that IDO1 knockout in myeloid cells increased ROS levels to aggravate graft-versus-host disease (Ju et al., 2021); however, whether IDO1 could suppress ROS production in tumors remains unclear. MDSCs also limit T-cell activation by consuming cystine in the TME (Srivastava et al., 2010).

##### Cystine depriving

It is well-known that cysteine is an essential amino acid for T-cell activation. Because of the lack of cystathionase and an intact Xc^−^ transporter, T cells cannot import cystine or convert intracellular methionine to cysteine(Ishii et al., 2004). Antigen presentation cells (APCs), including dendritic cells and macrophages, can import extracellular cystine and export cysteine to the TME, which is utilized by T cells. However, MDSCs import cystine but do not export cysteine because they do not express alanine-serine-cysteine transporters. Therefore, MDSCs competitively import cystine with APCs and reduce the level of cysteine in the TME to impair the uptake of T cells.

#### 3.1.4 Crosstalk Between MDSCs and Other Immune Cells

There are many other mechanisms that mediate T-cell suppression by MDSCs. Zheng et al. reported that the V-domain Ig suppressor of T-cell activation (VISTA) is highly expressed on MDSCs in the peripheral blood of patients with acute myeloid leukemia (Wang et al., 2018). VISTA knockdown in MDSCs impairs MDSC-mediated inhibition of CD8 T-cell activity. Recently, it was found that MDSCs could release a large number of exosomes to induce exhaustion and apoptosis of CD8^+^ T cells by increasing ROS production (Rashid et al., 2021).

##### NK cells

NK cells play important roles in anti-tumor immune response though directly killing tumor cells and indirectly activating Th1 immunity. Li *et al* reported that coculture with MDSCs induced anergy of NK cells, including decreased cytotoxicity, reduced production of IFN-γ, and downregulated expression of NKG2D (Li et al., 2009). They also found that the anergy of NK cells induced by MDSCs was dependent on membrane-bound TGF-β1, implying in a cell-cell contact manner. Additionally, another study showed that MDSCs could downregulate expression of CD247 on the surface of NK cells to inhibit their development and cytotoxicity (Vaknin et al., 2008).

##### DCs

As one of professional antigen-presenting cells (APCs), DCs are vital in adaptive immunity to suppress tumor progression. Emerging evidences have shown that MDSCs-DCs crosstalk contributed to DCs dysfunction. In tumor-bearing mouse model, the number of mature DCs decreased proportionately to the increasing number of MDSCs, which might be due to their competitively differentiation from common progenitor cells (Sinha et al., 2007). In addition, MDSCs might inhibit DCs maturation to induce immune tolerance and evasion. It has been reported that MDSCs-producing VEGF and IL-10 could downregulate expression of major histocompatibility complex (MHC) II and co-stimulators on DCs by activating STAT3 signaling (Heim et al., 2014). In another study, researchers found that MDSCs could suppress the process of antigen capture and the migration of immature DC to secondary lymphoid organs, which were essential for DCs maturation (Greifenberg et al., 2009). Moreover, MDSCs have been reported to alter cytokine production of DCs, including decreased secretion of IL-12 and increased secretion of IL-23 (Hu et al., 2011). These alterations might transform DCs from anti-tumor cells into pro-tumor cells by driving the proliferation and inflammatory function of Th17 cells (Langrish et al., 2005).

##### Macrophages

Macrophage are also one of the professional APCs to facilitate Th1 cells-mediated anti-tumor immunity. MDSCs have been demonstrated to alter the cytokine production, phenotype and antigen-presenting capacity of macrophages. It has been reported that IL-10 produced by MDSCs could decrease the secretion of IL-6, IL-12, and tumor necrosis factor-α (TNF-α) of macrophages, which remarkably suppress their anti-tumor activity (Beury et al., 2014). Moreover, Rosenberg *et al* found that MDSCs might switch macrophages phenotype from anti-tumor M1 subtype into pro-tumor M2 subtype, giving rise to so-called “tumor associated macrophages” (TAMs) (Ostrand-Rosenberg et al., 2012). Additionally, coculture with MDSCs could downregulate expression of MHC II molecular to interfere with antigen-presenting process of macrophages (Shin et al., 2006), resulting in immune tolerance or immune evasion.

##### Treg cells

Treg cells are also potent suppressor of T cells proliferation and cytotoxicity. As mentioned above, IL-10 and TGF-β could induce generation and expansion of Treg cells through increasing expression of CD25 and Foxp3. In addition, a series of chemokines produced by MDSCs, such as CCL3, CCL4, and CCL5, could promote recruitment of Treg cells into TME.

### 3.2 Non-immunological Functions of MDSCs in Tumor Progression

In general, tumor cells produce certain growth factors and chemokines to recruit MDSCs into the TME. In addition to suppressing T-cell-mediated immunity, MDSCs promote tumor progression and metastasis by mediating stemness of tumor cells, angiogenesis, and degradation of the extracellular matrix (ECM).

#### 3.2.1 Effects on EMT and Stemness

Numerous studies have indicated that MDSCs mediate the epithelial-to-mesenchymal transition (EMT) of tumor cells in many cancers (Zhu H et al., 2017; Pang et al., 2020). EMT is a critical biological process in the formation of cancer stem cells (CSCs), which harbor stem cell properties such as self-renewal, multi-lineage differentiation, and tumorigenicity (Dave et al., 2012). Many immunosuppressive factors produced by MDSCs, such as TGF-β (Katsuno et al., 2013), VEGF (Oka et al., 2007), IL-10 (Yang et al., 2019), and IL-6 (Kim et al., 2013), have been shown to induce EMT and stemness in various tumor cells. Liu et al. reported that CXCR2^+^ MDSCs induce EMT in breast cancer cells by secreting IL-6 (Zhu H et al., 2017). In a melanoma model, MDSCs promoted EMT via TGF-β, epidermal growth factor, and hepatocyte growth factor signaling pathways (Schlegel et al., 2015). In 2013, Zou et al. first reported the interaction between MDSCs and CSCs (Cui et al., 2013). They found that MDSCs triggered miRNA101 expression in ovarian cancer cells to repress the co-repressor gene C-terminal binding protein-2 (CtBP2), which directly targets stem cell core genes, resulting in increased cancer cell stemness and increased metastatic and tumorigenic potential. Another study indicated that MDSCs increased ALDH^high^ CSCs in ovarian cancer by producing PEG2. Moreover, PEG2 produced by MDSCs upregulated PD-L1 expression in ovarian cancer cells (Komura et al., 2020). Linehan et al. demonstrated that MDSCs significantly increased the frequency of ALDH1^Bright^ CSCs in a mouse model of pancreatic cancer through activation of the STAT3 pathway (Panni et al., 2014). In addition to inducing EMT and stemness, MDSCs upregulate caspase-1 to directly promote the proliferation of multiple squamous carcinoma cells *in vitro* and *in vivo* (Zeng et al., 2018).

#### 3.2.2 Effects on Angiogenesis

MDSCs have been proven to facilitate angiogenesis in tumor progression through many different mechanisms. MDSCs produce high levels of VEGF (Shojaei et al., 2009), which is the most important cytokine involved in angiogenesis. VEGF binds to its receptor (VEGFR) on adjacent epithelial cells to promote neovascularization by activating the JAK2/STAT3 pathway. Interestingly, MDSCs in TME also express high levels of VEGFR2 (Min et al., 2017), which may be activated by VEGF secreted by tumor cells or themselves to produce more VEGF. Therefore, the VEGF/VEGFR pathway establishes a positive feedback loop in MDSCs to maintain their angiogenic activity. Moreover, MDSCs are able to produce an abundance of proteolytic enzymes, such as matrix metalloproteinases (MMPs) (Zhang et al., 2020), which are involved in tumor metastasis by degrading the ECM. MMPs are also key regulators of angiogenesis. Yang et al. reported that MMP9 was highly secreted by Gr-1^+^CD11b^+^ MDSCs, and that deletion of MMP9 decreased vascular density, vascular maturation, and tumor growth (Yang et al., 2004). Additionally, other factors contribute to MDSC-induced angiogenesis, including Bombina variegata peptide 8 (Bv8), TGF-β, fibroblast growth factor 2, and IL-1β. Ferrara et al. reported that Gr-1^+^CD11b^+^ myeloid cells upregulated the expression of Bv8 to promote tumor angiogenesis (Shojaei and Ferrara, 2008). As mentioned, MDSCs can produce TGF-β. TGF-β induces tumor angiogenesis by activating fibroblasts to produce ECM adhesion and stimulating tube formation by endothelial cells (van Meeteren et al., 2011). Further studies have indicated that TGF-β may mediate acquired resistance to anti-VEGF therapy (Aguilera et al., 2014). Yu et al. reported that STAT3-activated MDSCs upregulated the expression of basic fibroblast growth factor 2 and IL-1β (Kujawski et al., 2008), which are critical for tumor-derived MDSC–mediated angiogenesis.

Some RNA contents of MDSC-derived exosomes also contribute to tumor angiogenesis. Zhang et al. showed that MDSC miR-126a rescues doxorubicin-induced MDSC death in a S100A8/A9-dependent manner and promotes tumor angiogenesis (Deng et al., 2017). In another study, miR-9 contained in MDSC-derived exosomes was found to promote tumor angiogenesis by reprogramming endothelial cells (Baroni et al., 2016). However, other exosomes involved in MDSC-mediated angiogenesis require further study.

#### 3.2.3 Effects on Pre-metastatic Niche Formation

Clinical data have proven that MDSCs positively correlated with distant metastasis in various tumors, including NSCLC, breast cancer, melanoma, prostate cancer and so on (Huang et al., 2013; Yu et al., 2013; Weide et al., 2014; Hossain et al., 2015). Besides the promotion of EMT and angiogenesis, MDSCs have been demonstrated to the formation of pre-metastatic niche. Wang et al. reported that CXCL1 produced by TAMs in tumor microenvironment could recruit CXCR2^+^ MDSCs to liver and establish a pre-metastatic niche that expedited liver metastasis (Wang et al., 2017). In another mouse model of breast cancer, CCL2 secreted by tumor cells could recruit MDSCs to the lung and result in formation of pre-metastatic niche of lung metastases (Sceneay et al., 2013). These functions might be explained by the degradation of the extracellular matrix (ECM) to make the local microenvironment more permissive for the seeding of circulating tumor cells (Wang et al., 2019b). Moreover, MDSCs-produced cytokines and chemokines, such as TGF-β, VEGFA, S100A8/A9, and MMP-9, might play important role in pre-metastatic niche formation through facilitating angiogenesis and tumor cell invasion (Shojaei et al., 2009).

## 4 MDSCs Act as a Negative Biomarker of Immunotherapy

The emergence of immunotherapy has achieved a great breakthrough in cancer therapy. In particular, T cell-based immunotherapy such as immune checkpoint blockers (ICBs), and the infiltration and function of cytotoxic T lymphocytes (CTLs) directly determines the efficacy of immunotherapy. Accumulating evidence has shown that MDSCs impair the patient response and survival, to clarify the role of MDSCs in immunotherapy has profound significance.

Several clinical studies have identified MDSCs as predictive biomarkers of poor prognosis for immunotherapy in many cancers. It has been reported that the responders to ipilimumab have lower frequency of MDSCs in peripheral blood than the non-responders in patients with melanoma (Meyer et al., 2014). The result of IMmotion150 identified that high myeloid inflammation gene signature expression (Myeloid^High^) was associated with reduced PFS in atezolizumab-treated patients with metastatic renal cell carcinoma (mRCC) (McDermott et al., 2018). Moreover, MDSCs have been shown to predict the efficacy of anti-PD-1 immunotherapy. Youn et al. demonstrated that the ratio of Treg cells to Lox-1^+^ MDSCs was positively correlated with anti-PD-1 efficacy in non-small cell lung cancer (NSCLC) (Kim et al., 2019). Another study found that the ratio of NK cells to Lox-1^+^ PMN-MDSCs was also an ideal biomarker for PD-1 antibody treatment of NSCLC (Youn et al., 2020). Recently, low frequency of MDSCs in the peripheral blood has been identified to be associated with longer progression free survival and overall survival in NSCLC patients after PD-1 treatment (Koh et al., 2020). Therefore, MDSCs might be a potential tool for cancer treatment or for improving the efficacy of immunotherapy.

## 5 Strategies Targeting MDSCs for Optimizing Cancer Treatment

To date, several clinical trials have been established to specifically target MDSCs to inhibit proliferation, decrease the expression of immunosuppressive mediators, block recruitment, and promote differentiation.

### 5.1 Blockade of MDSCs’ Recruitment

MDSCs express high levels of certain chemokine receptors, such as CXCR1, CXCR2, CXCR4, CCR2, and CCR5. Therefore, MDSCs can be recruited into the TME by corresponding chemokines from tumor cells and immune cells, including CXCL5, CXCL8, CXCL12, CCL2, and CCL5. Many preclinical and clinical trials have attempted to block MDSC recruitment with antagonists of these chemokine receptors. In 2016, a phase Ib study investigated the safety and efficacy of PF-04136309 (a CCR2 antagonist) and FOLFIRINOX for locally advanced and borderline resectable pancreatic cancer (Nywening et al., 2016). The results showed that the ORR and DCR for the combined group were 49 and 97%, respectively, but they were only 0 and 80%, respectively, for the chemotherapy alone group. Although the authors speculated that the regression of tumors was attributable to decreased TAMs by CCR2 blockade, the contribution of reduced MDSCs cannot be ignored. Another CCR2 antagonist, 747, has been proven to exhibit anti-cancer properties alone and potentiate the anti-tumor efficacy of sorafenib in a hepatocellular carcinoma model (Yao et al., 2017); however, further clinical studies are needed. A phase Ib study was established by Goldstein et al. to investigate the efficacy of the CXCR1/2 inhibitor, reparixin, in combination with weekly paclitaxel for metastatic HER2 negative breast cancer (Schott et al., 2017). They reported an ORR of 27.8% (5/18 patients). CXCR4 antagonists and antibodies are designed to treat acute myeloid leukemia, multiple myeloma (MM), and solid tumors. Thornton et al. published the results of a phase I trial of LY2510924, a CXCR4 peptide antagonist, for patients with solid tumors (Galsky et al., 2014). For the 45 patients in this trial, the DCR was 20% and the ORR was not observed. Phase II and phase III trials are required to enroll more patients. Recently, the efficacy of a CXCR4 antibody, ulocuplumab (BMS-936564), in combination with lenalidomide or bortezomib plus dexamethasone for MM was studied. The results showed that the ORR of combined therapy was 55.2% and the DCR was 72.4% (Ghobrial et al., 2020).

### 5.2 Inhibition of MDSCs’ Activation

Inducing immature MDSCs into mature myeloid cells may be a potential strategy for treating cancer. All-trans retinoic acid (ATRA) has been recognized as an ideal inducer of differentiation and has been widely used for many hematopoietic tumors (Mirza et al., 2006; Iclozan et al., 2013). Moreover, McCarter et al. found that ATRA could decrease the frequency and suppressive functions of MDSCs and increase the frequency of circulating mature myeloid cells in the peripheral blood of patients with melanoma (Tobin et al., 2018). However, the efficacy of ATRA alone or in combination with ipilimumab for melanoma patients requires further study. Furthermore, 1,25 dihydroxy vitamin D (1,25(OH)_2_D), the active form of vitamin D, is involved in cell differentiation. The use of 1,25(OH)_2_D has been proven to block tumor progression and prolong disease-free survival by increasing CD4^+^ and CD8^+^ T-cell infiltration in patients with head and neck squamous cell carcinoma (Makitie et al., 2020). Other studies reported that MDSCs in the TME expressed high levels of vitamin D receptor, and that 1,25(OH)_2_D treatment could significantly inhibit their suppressive functions (Fleet et al., 2020). However, the effects of 1,25(OH)_2_D on MDSC differentiation require further study.

### 5.3 Depletion of MDSCs’ Expansion

The CSF-1/CSF-1R pathway has a key role in MDSC proliferation, and CSF-1R inhibitor treatment has resulted in exciting results. Tyner et al. identified GW-2580, a CSF-1R inhibitor, as a potential anti-tumor agent for acute myeloid leukemia by decreasing CD33^+^ MDSC infiltration (Edwards et al., 2019). Imatinib, a TKI approved for the treatment of chronic myeloid leukemia and gastrointestinal stromal tumors, was also found to target GSF1R. Imatinib has been shown to significantly reduce the frequency of circulating G-MDSCs in patients with chronic myeloid leukemia (Giallongo et al., 2018). In 2019, Rugo et al. published the results of a phase Ib study of the combination of pexidartinib (another CSF-1R inhibitor) and paclitaxel for patients with advanced solid tumors (Wesolowski et al., 2019). They found that of 38 evaluable patients, 1 (3%) had a complete response, 5 (13%) had a partial response, and 13 (34%) had stable disease. The overall response rate (ORR) and disease control rate (DCR) were 16 and 55%, respectively. They also found that combined therapy reduced CD14^dim^/CD16^+^ monocyte levels by 57–100% in peripheral blood; however, the effect on MDSCs was not studied further.

### 5.4 Target on MDSCs’ Metabolic Products

As mentioned, Arg-1 and iNOS are critical factors in MDSC-mediated immune suppression. A phase I study of the arginase inhibitor INCB001158 (1,158) alone and in combination with pembrolizumab for patients with solid tumors revealed that the ORR and DCR for the 1,158 alone group and the combined group were 3 and 28% and 6 and 37%, respectively (Naing et al., 2019). Recently, a phase I/II study of INCB001158 plus chemotherapy for patients with advanced biliary tract cancers reported that the ORR was 24% (8/33 patients; 95% confidence interval [CI], 11.1–42.3%), the DCR was 67%, and the median progression-free survival was 8.5 months (95% CI, 5.7–10.1 months) (Javle et al., 2021). Similarly, a phase I/II clinical trial performed to study the efficacy of the iNOS inhibitor NG-monomethyl-L-arginine (L-NMMA) combined with docetaxel treatment reported that the ORR was 22.2% for patients with triple-negative breast cancer (Chung et al., 2019). These results suggest that inhibitors of Arg-1 and iNOS combined with immunotherapy or chemotherapy might be better choices for some solid cancers (Table 1).

**TABLE 1:** Strategies targeting MDSCs.

Mechanism	Manner	Agents	Type of cancer	Outcomes	Ref
Blockade of recruitment	CCR2 antagonist	PF-04136309	Pancreatic cancer	ORR: 49% DCR: 97%	Nywening et al. (2016)
	CCR2 antagonist	747	Hepatocellular carcinoma	NA	Yao et al. (2017)
	CXCR1/2 inhibitor	Reparxin	Breast cancer	ORR:27.8%	Schott et al. (2017)
	CXCR4 antagonist	LY2510924	Pan-solid cancer	DCR: 20%	Galsky et al. (2014)
	CXCR4 antibody	Ulocuplumab (BMS-936564)	Multiple myeloma	ORR:55.2% DCR: 2.4%	Ghobrial et al. (2020)
Deplete and inhibit activation	Vitamin A	ATRA	Small cell lung cancer	ORR: 41.7%	Mirza et al. (2006)
	Vitamin D	1,25(OH)_2_D	Head and neck cancer	NA	Makitie et al. (2020)
Inhibit expansion	CSF-1R inhibitor	GW-2580	Acute myeloid leukemia	NA	Edwards et al. (2019)
	CSF-1R inhibitor	Imatinib	Chronic myeloid leukemia	NA	Giallongo et al. (2018)
	CSF-1R inhibitor	Pexidartinib (Ib)	Solid cancer	ORR: 16% DCR: 55%	Wesolowski et al. (2019)
Target on metabolic products	Arg-1 inhibitor	INCB001158 plus pembrolizumab (I)	Solid cancer	ORR: 28% DCR: 37%	Naing et al. (2019)
	Arg-1 inhibitor	INCB001158 (I/II)	Biliary tract cancers	ORR: 24% DCR: 67% mPFS: 8.5m	Javle et al. (2021)
	iNOS inhibitor	L-NMMA	Triple-negative breast cancer	ORR: 22.2%	Chung et al. (2019)

In fact, reductions in MDSC frequency and function have been demonstrated during regular anti-tumor therapies, including chemotherapy, targeted therapy, immunotherapy, and combined therapy. However, other studies and our previous studies have shown that radiotherapy could induce an increase in MDSCs in the TME and spleen (van Meir et al., 2017; Cheng et al., 2021). Regarding chemotherapeutic agents, 5-fluorouracil (5-FU), gemcitabine, and pemetrexed could decrease MDSC accumulation in various cancers. However, Baniyash et al. reported that CPT-11 (irinotecan) increased the abundance and immunosuppressive features of MDSCs in colorectal cancer by blocking MDSC apoptosis and myeloid cell differentiation (Kanterman et al., 2014). Anti-angiogenic drugs, such as bevacizumab and some small molecular tyrosine kinase inhibitors (TKIs), can also reduce MDSC accumulation. Kotsakis et al. found that bevacizumab-based chemotherapy significantly reduced the percentage of MDSCs compared with non-bevacizumab regimens in the peripheral blood of patients with NSCLC (Koinis et al., 2016). In a previous study, nilotinib, dasatinib, and sorafenib, but not sunitinib, inhibited the differentiation of MDSCs and promoted their immunosuppressive capacity (Heine et al., 2016). However, in a model of mRCC, both sunitinib alone and in combination with PD-1 antibody significantly reduced a large proportion of G-MDSCs and M-MDSCs (Rayman et al., 2015). And another study also found that sunitinib treatment reduces intratumoral MDSC content, especially significantly shrinkage of G-MDSC in RCC patients (Guislain et al., 2015). Aerts et al. found that axitinib with αCTLA-4 increased the number of MDSCs but decreased their suppressive capacity in a melanoma brain metastasis model (Du Four et al., 2016). Olaparib, targeting poly (ADP-ribose) polymerase (PARP), also suppressed MDSC recruitment by dampening the CXCL12/CXCR4 pathway (Sun et al., 2021). Although epidermal growth factor receptor TKIs, including gefitinib and osimertinib, were found to increase the level of MDSCs in NSCLC (Jia et al., 2019), whether their suppressive functions are modified requires further study. Additionally, immune checkpoint inhibitors (anti-PD-1, anti-PD-L1, and anti-CTLA-4) have been reported to reduce the frequency of MDSCs and decrease the production of suppressive factors and chemokines. Grivas et al. found that PD-L1 antibody (atezolizumab/avelumab) and PD-1 antibody (pembrolizumab) were correlated with a decreased percentage of PD-L1^+^ MDSCs in patients with metastatic urothelial carcinoma (Tzeng et al., 2018). Kiessling et al. reported that ipilimumab, a CTLA-4 antibody, reduced the infiltration of MDSCs and iNOS expression in melanoma patients (de Coana et al., 2017).

## 6 Conclusion and Perspectives

Emerging evidence has implied the suppressive role of MDSCs in pathological conditions including tumor progression. Although we have a better knowledge of the origin and development of MDSC, the genomic and metabolic differentiation mechanisms are still unclear. Recent advances in genomic of high-resolution and metabolic technologies may allow for further study development and differentiation. In consideration of the pro-tumor contribution of MDSCs from immunological and non-immunological view in tumor progression and metastasis just as we have discussed in this review, to clarify their role in anti-tumor treatment especially immunotherapy is particularly important. In clinic, MDSCs are negative prognostic biomarkers for multiple tumors, and may mediate acquired resistance to immunotherapy. Although many clinical trials have attempted to target MDSCs for improving clinical benefit, their efficacy is far from our expectations. The major obstacle is to understand the complex mechanisms and target MDSCs selectively. Therefore, more clinical studies and basic research involving targeting MDSCs alone or in combination with immunotherapy are needed.
